# New novel thermal insulation and sound-absorbing materials from discarded facemasks of COVID-19 pandemic

**DOI:** 10.1038/s41598-021-02744-8

**Published:** 2021-12-01

**Authors:** M. Ali, R. Almuzaiqer, K. Al-Salem, A. Alabdulkarem, A. Nuhait

**Affiliations:** 1 Mechanical Engineering Department, College of Engineering, King Saud University, PO Box 800, Riyadh, 11421 Saudi Arabia; 2 K.A.CARE Energy Research and Innovation Center at Riyadh, Riyadh, Saudi Arabia

**Keywords:** Environmental sciences, Energy science and technology, Engineering, Materials science

## Abstract

Due to the COVID-19 pandemic, people were encouraged and sometimes required to wear disposable facemasks, which then are discarded creating an environmental problem. In this study, we aim at investigating novel ideas to recycle wasted facemasks in order to lower the environmental impact. An experimental study has been carried out to investigate the possibility of using discarded masks for thermal insulation and sound absorption. The wasted masks are simulated by new masks, which stripped off the nose clips, elastic ear loops and are heated to 120 °C for one hour to kill any biological contaminants. The masks are also melted to investigate their thermal insulation and sound absorption properties. Results show that the thermal conductivity coefficients of the loose and melted masks are 0.03555 and 0.08683 W/m K, respectively, at room temperature of about 25 °C. Results show also that the sound absorption coefficient for loose masks is above 0.6 for the frequency range 600–5000 Hz. The loose facemasks are found to be thermally stable up to 295 °C, elastic ear loops at 304.7 °C, and the composite (melted) facemasks at 330.0 °C using the thermo-gravimetric analysis. Characterization of the facemask’s three-layer fibers and the composite (melted) samples is obtained using scanning electron microscopy (SEM). The three-point bending test is obtained for the composite specimens showing good values of flexural stress, flexural strain, and flexural elastic modulus. These results are promising about using such discarded masks as new thermal insulation and sound-absorbing materials for buildings replacing the synthetic or petrochemical insulation materials.

## Introduction

During the pandemic COVID-19 era, huge numbers of disposable facemasks are used for personal protection. The health ministries in most countries around the world have mandated or recommended the wearing of facemasks in public settings. Eighty-nine million medical masks were required each month to cover the COVID-19 pandemic response^1^. Nzediegwu and Chang^2^, have shown that the estimated daily facemask used in some African states reached seven hundred million. The majority of masks are single-use and have to be discarded afterward causing an already existing waste problem to grow worst. Sharma et al.^3^ have highlighted some innovative management challenge solutions to the biomedical existing waste during the crisis brought upon by the COVID-19 pandemic. Serin and Caglar^4^ have investigated the effect of different masks used as personal protective equipment on resuscitation quality and rescuer fatigue. They have concluded that protective masks other than surgical masks used as personal protective equipment increased rescuer fatigue in Cardiopulmonary resuscitation and negatively affected the quality of chest compressions. The simplest method to limit the spread of the COVID-19 pandemic and to protect the people is to wear a facemask in crowded places. However, manufacturers may have a problem providing enough facemasks in a short time. Therefore, Phan and Ching^5^ have shown an idea on how to save the number of used facemasks but still provide the same protective values using a Cardiopulmonary resuscitation mask and a common surgical facemask. Their suggested method should be applied where people cannot afford to have enough surgical masks to protect themselves. Analytic hierarchy process was used by Hartanto and Mayasari^6^ to determine the appropriate materials for making environmentally friendly non-medical masks. Zorko et al.^7^ have reviewed the possibility of reusing the surgical medical masks. They were unable to conclude on the most efficient and safe process for decontaminating surgical masks. Côrtes et al.^8^ have shown that the most simple and useful method to decontaminate surgical masks was by using dry heat in the oven at 75 °C for 45 min. It was also found by the National Health Commission of the People’s Republic of China^9^ that, the temperature of 56 °C for 30 min was enough for effectively inactivating the COVID-19 virus. Liao et al.^10^ have found that heating ≤ 85 °C under various humidities was the most promising, nondestructive method for disinfection while preserving the filtration properties of the mask. It was found that soaking the used masks in hot water at temperatures > 56 °C for 30 min is enough to kill COVID-19 virus^11^. Abraham et al.^12^ have shown that heating for 3 min at 75 °C, 5 min at 65 °C, or 20 min at 60 °C was enough to kill the COVID-19 virus. On the other hand, Saberian et al.^13^ have shown that blends of one percent of the shredded facemasks added to the recycled concrete aggregate resulted in the highest values of unconfined compressive strength and the highest resilient modulus. Their new recycled concrete aggregate blended with shredded facemasks could be used for road base and subbase applications. Rehman and Khalid^14^ have reported a novel soil treatment method for improving the mechanical characteristics of fat clay by using facemask as fiber reinforcement and silica fume as the cementitious agent in the form of a composite binary admixture. They observed that composite binary admixture improved the strength characteristics more than silica fume but also regulated the ductility and deformability of the treated soil due to the presence of facemask fibers. Lynch et al.^15^ have used cut-up facemask in concrete construction at different percent. They have found that introducing the facemasks below 20% increased both the strength properties of the concrete samples and the overall quality of the concrete. Asim et al.^16^ have conducted a review study about the potential practices for waste management related to waste valorization of discarded face masks as the major type of waste during the COVID-19 pandemic. Ali et al.^17–21^ have presented new thermal insulation materials extracted from some different agro materials, which were considered as waste. Their study included palm tree surface fibers and leaves, wheat straw, agave fibers, and Eucalyptus Globulus leaves. A hybrid of these materials has been developed and lab-scale samples were tested toward their thermal insulation and sound-absorbing. Ali and zeitoun^22^ and Ali^23^ have reported the use of Apple of Sodom fibers as a new source of insulation and sound-absorbing. They used the cornstarch as a binder for the fibers, which shows good thermal conductivity coefficients approaching that of the ASTM standard.

This paper presents an experimental study to investigate a novel idea for recycling discarded facemasks by reusing them as thermal insulation and sound absorption materials. Both loose and melted facemasks are investigated in this study. Acoustic analysis of the loose and melted masks is presented for a wide range of frequencies. Thermal conductivity coefficients are obtained for a wide range of temperatures for both loose and melted facemasks. Thermal analysis of the facemask’s fibers and their composite is reported as well as the bending moment of the composite specimens. The surface morphology of the masks is discussed. It should be noted that all the images that appear in this study were taken by the authors and all the tests and measurements were taken in our mechanical engineering lab at our university "King Saud University".

## Materials and experimental methods

### Investigated facemasks

**Figure 1:**
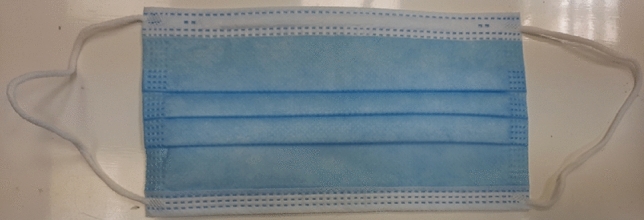
Ear loop facemask used in the current study.

Unused disposable facemasks with ear loop type are used in this study as shown in Fig. 1. The main components of the mask are a three-layer structure (The middle layer, which is the filter media, the absorbing moisture inner layer (white layer # 3), and the repel water outer layer (blue layer # 1), the metal nose clip, and elastic ear loops. The used mask size is 17.5 × 9.5 cm^2^ and manufactured by Foshan RongFang YouPin Textile Co., Ltd, China.

### Preparation of loose samples

**Figure 2:**
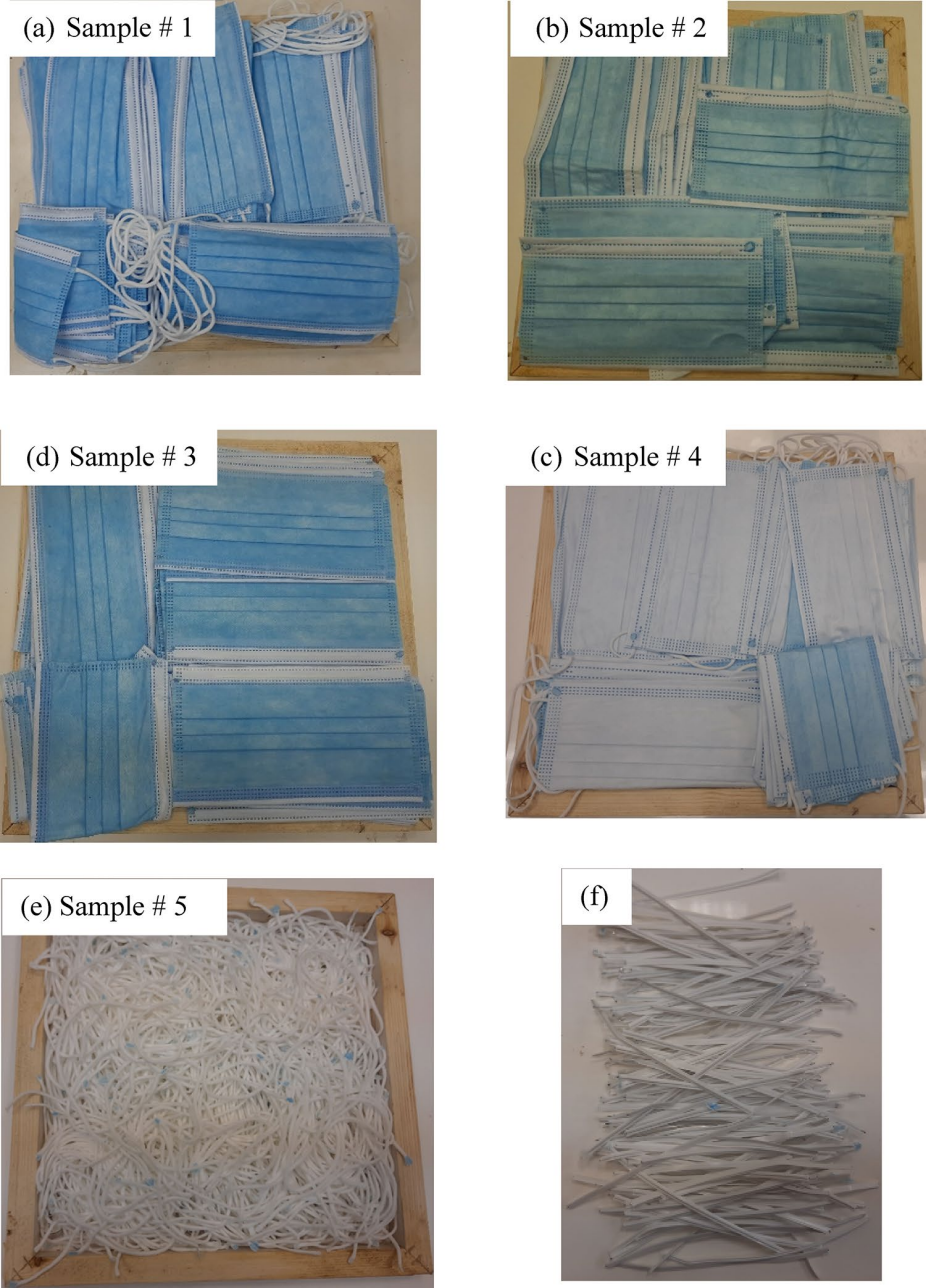
Prepared loose samples; (**a**) complete masks, (**b**) masks with removed both of metal nose clip and elastic ear, (**c**) masks with metal nose clip but removed elastic ear loops, (**d**) masks with elastic ear loops but removed metal nose clip, (**e**) elastic ear loops, and (**f**) removed metal nose clip.

To simulate the used and may be infected facemasks, new unused samples of facemasks are heated in a convection oven at 120 °C for one hour to simulate the viral disinfection. However, all precautions must be taken when collecting infected real discarded facemasks. Five samples are prepared for the thermal conductivity measurements as shown in Fig. 2a–e. The whole facemask (Fig. 2a), masks without metal nose clip or elastic ear loops (Fig. 2b), masks with nose clip only (Fig. 2c), masks with elastic ear loops only (Fig. 2d), and loose elastic ear loops only (Fig. 2e).

Figure 2f shows the removed metal nose clip. Table 1 lists the physical properties and densities of the samples shown in Fig. 2.

**Table 1:** Physical properties of the tested samples.

Sample no.	1	2	3	4	5	6	7
Material	Complete face masks	Masks without metal nose clip or elastic ear loops	Mask with metal nose clip only	Masks with elastic ear loops only	Loose elastic ear loops only	Melted masks with metal nose clips	Melted masks without metal nose clips
Fig. no.	2(a)	2(b)	2(c)	2(d)	2(e)	5(a)	5(b)
Mass (g)	421	154	217	183	134	566	487
Thickness (cm)	4.24	2.44	2.94	2.65	2.10	1.12	0.79
Size (cm^2^)	24 × 24	24 × 24	24 × 24	24 × 24	24 × 24	29 × 29	29 × 29
Density (kg/m^3^)	172.4	109.6	128.0	120.0	110.8	601.0	733.0
Symbol	▲	+	□	■	◇	●	○

### Preparation of melted samples

**Figure 3:**
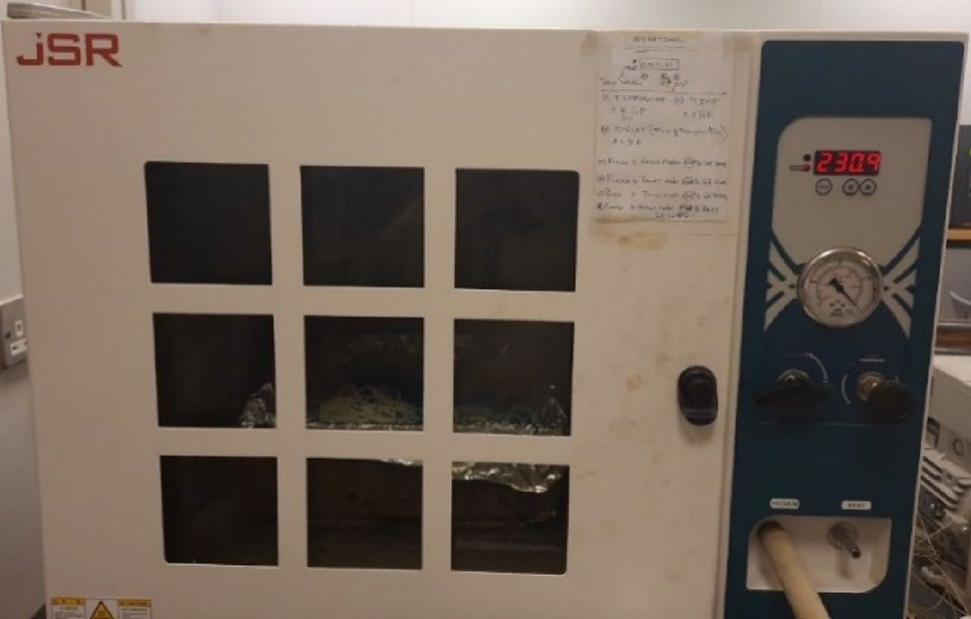
The used convection oven showing the melting temperature.

**Figure 4:**
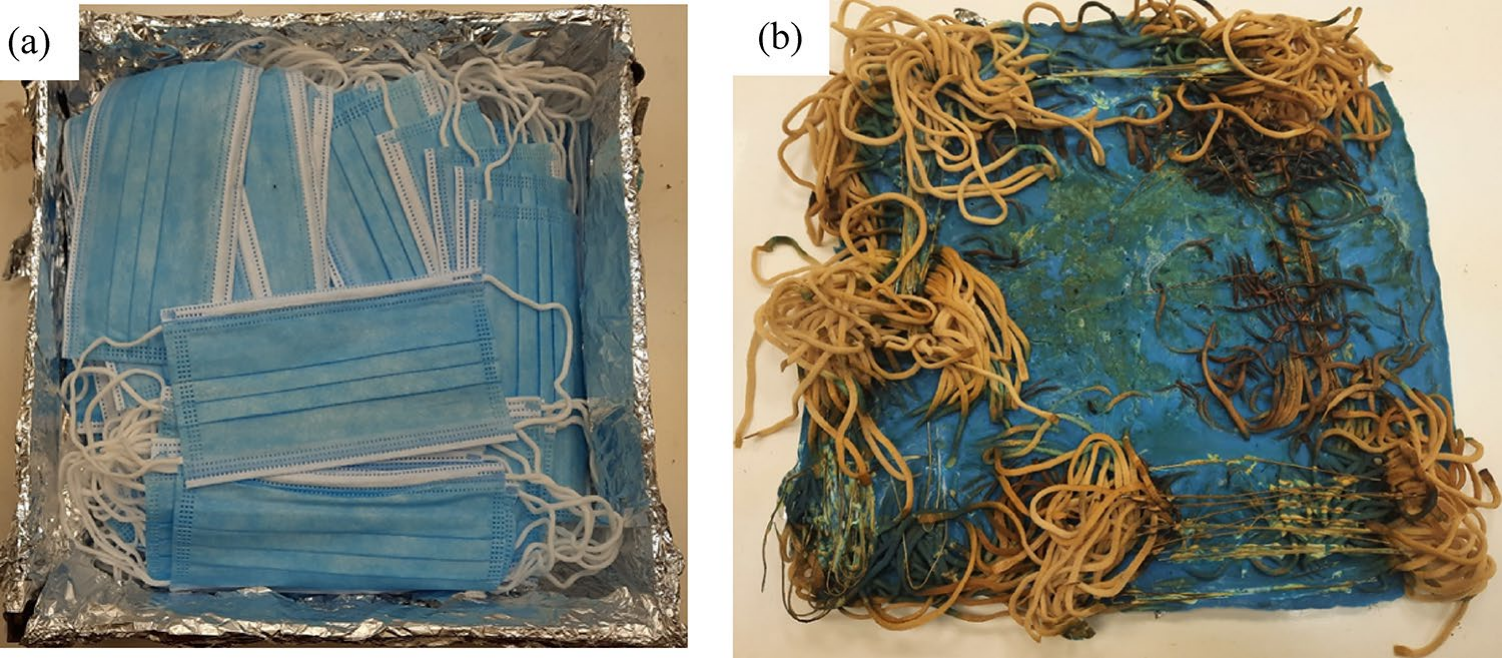
(**a**) The stainless steel mold holding the masks before melting and (**b**) the melted masks showing the unmelted elastic ear loops.

**Figure 5:**
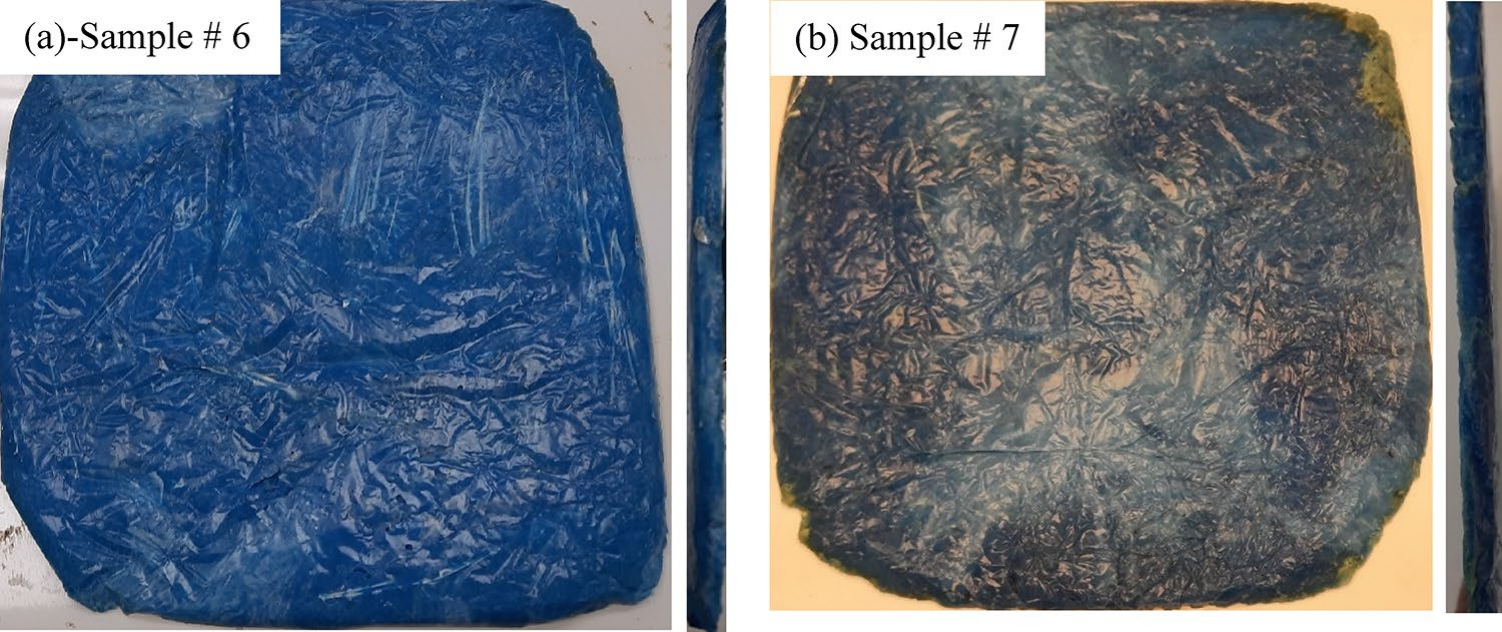
Melted masks (**a**) with metal nose clips and (**b**) without metal nose clips.

The whole sample masks are melted at 230 °C in a convection oven (Fig. 3). A stainless steel mold (Fig. 4a) was used to hold the samples. The ear loops were excluded because they needed higher temperature to melt, Fig. 4b, and the samples were melted with and without the metal nose clips. Figure 5a, b shows the melted masks with and without metal nose clips. Specifications of melted samples are shown in Table 1.

## Fiber and composite characterization

Characterization of the facemask’s three-layer fibers and the composite (melted) samples is obtained using scanning electron microscopy (SEM). Furthermore, thermal stability analysis for the mask’s fibers and composite is obtained.

### Scanning electron microscopy (SEM) analysis

Field emission scanning electron microscope (FE-SEM) (JEOL; JSM7600F) was used to examine the surface morphology of the masks’ three-layer fibers and their composite (melted) samples at different magnifications. Before performing the test, the samples were dried in an oven and coated with Platinum under vacuum. This step is mandatory to avoid any electrostatic charging, which may happen during the test. The analysis of this instrument follows the ASTM-E1508-98^24^ Standard.

### Thermal stability analysis

Thermal stability and decomposition of the mask’s layer fibers and their composite ones are obtained by performing the thermo-gravimetric (TGA), the differential (DTGA) analyses, and the differential scanning calorimetry (DSC). The TGA analysis is conducted with an SDT Q600 V20.9 Build 20 setup from TA instruments (U.S.) fitted with nitrogen purge gas. Sample masses of 3.85 mg, 2.02 mg, and 3.34 mg of elastic ear loops, mask’s three-layer fibers, and the composite (melted without ear loops or metal nose clips), respectively are used for the analyses. Each sample is kept in an alumina pan and heated to 600 °C starting from room temperature (25 °C), at a heating rate of 10 °C/min, and with a nitrogen gas flow of 100 mL/min.

## Thermal conductivity measurements

Heat flow meter (HFM 436 Lambda, bench type) is used to measure the thermal conductivity of both loose facemasks (Fig. 2, samples 1–5) and composite (melted) (Fig. 5, samples 6–7) samples in the temperature range of 10–70 °C. Each sample is sandwiched between a hot and a cold plate, and the heat flow created by the well-defined temperature difference (20 °C) is measured with a heat flux sensor. The HFM is a calibrated instrument^25^, which performs tests according to ASTM-C518^26^ standards and uses a standard sample size of 30 × 30 cm^2^ with a variable thickness up to 10 cm. The HFM comes with an integrated μm-resolution transducer, allowing the measurement of the actual board’s thickness. Technical data provided by the manufacturer shows that all measurement results of the thermal conductivity are in agreement within 0.5% (error bars), the accuracy in reading the temperature and the thermal conductivity is ± 0.01 °C and ± 1 to 3% W/mK.

## Sound absorption coefficient measurements

**Figure 6:**
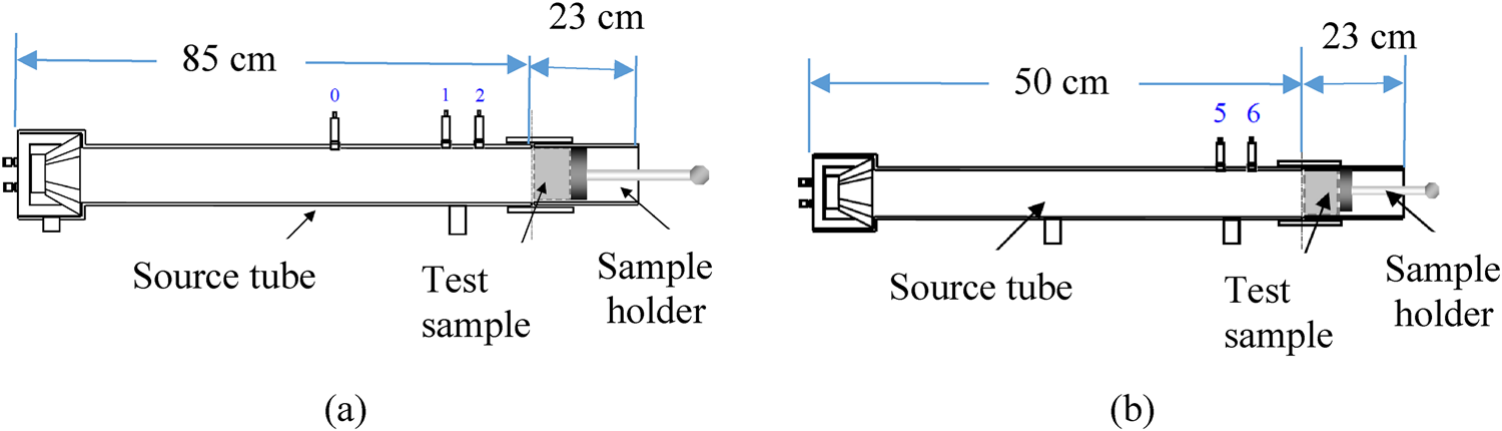
Specifications of the used impedance tubes; (**a**) 10 cm diameter with microphones number 0, 1, and 2 and (**b**) 3 cm diameter with microphone number 5 and 6.

The absorption acoustic coefficient was measured with an impedance tube. The principles of the impedance tube are based on plane waves generated by a loudspeaker and the sound pressures are measured with two microphones, while the samples are mounted opposite to the loudspeaker. The loudspeaker emits a sound (in this case a pink noise) in all frequency measurement range. The sound travels in the tube and when it meets the specimen, part of the sound is absorbed and the other part is reflected. With the union between the incident and the transmitted sound wave, a standing wave is formed whose sound pressure value is detected by the microphones. The difference in sound pressure measured between the two microphones provides the value of the quantity of sound energy absorbed by the measuring specimen. In this way, it is possible to calculate the value of the sound absorption coefficient in the frequency range. The measuring frequency range (lower and upper frequency) depends on the tube inside diameter and the spacing between the microphones. Two impedance tubes are used one with 10 cm inner diameter (Fig. 6a) and the other with 3 cm (Fig. 6b). The frequency range depends on the diameter of the impedance tube and the spacing between the two microphones. Consequently, if the 10 cm diameter tube (Fig. 6a) is chosen, then two options are available, i.e. either using microphone 1 and 2 for a frequency range of 400–1600 Hz or use microphone 0 and 2 for a frequency range of 63–500 Hz. On the other hand, if the 3 cm diameter tube (Fig. 6b) is chosen, then the possible range of frequency is 800–6300 Hz (as specified by the manufacturer: BSWA Tech.).

**Figure 7:**
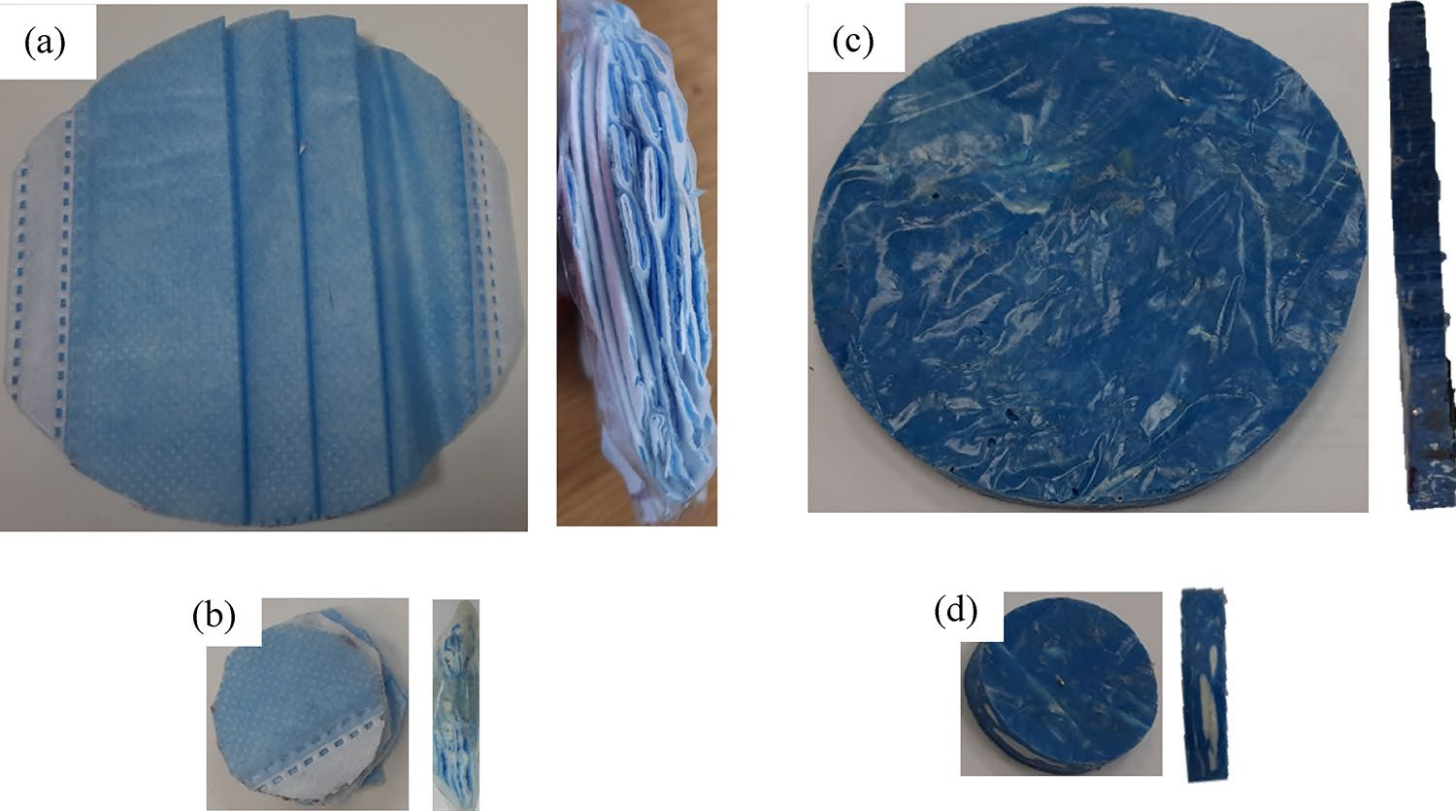
Sampled used for acoustic measurement; (**a**) 10 cm diameter loose masks with 12 mm thickness, (**b**) 3 cm diameter loose masks with 11 mm thickness, (**c**) 10 cm melted masks, and (**d**) 3 cm melted masks.

The test procedure with the impedance tube follows the standards (ISO 10534-1^27^ and ISO 10534-2^28^). These acoustic measurements were obtained for loose facemasks without elastic ear loops and either with or without metal nose clips and for melted samples 6 and 7. Figure 7 shows the samples used for acoustic measurements.

## Mechanical properties of the composite samples

**Figure 8:**
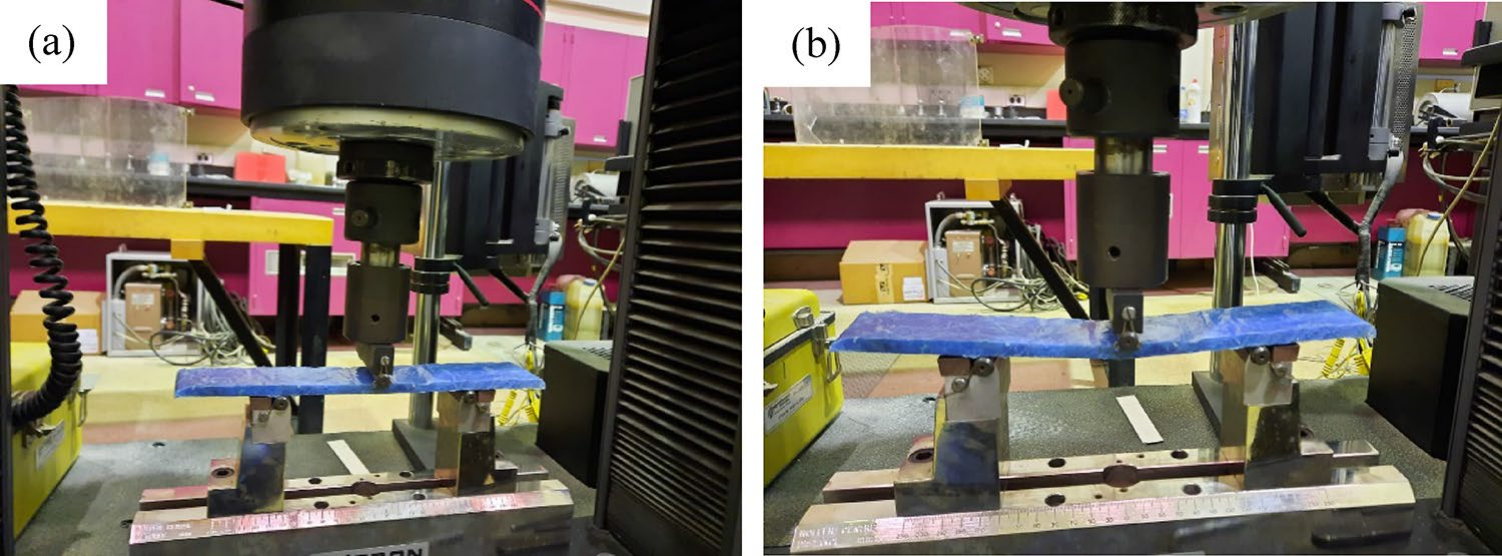
The Universal Testing Machine used for the three-point bending moment test; (**a**) before the test and (**b**) after the test.

A three-point bending moment test is obtained for the composite samples number 6 and 7. The Universal Testing Machine (Fig. 8) of crosshead speed of 2 mm/min (UTM, INSTRON 5984) is used for determining the load, deflection, flexural stress ${\sigma }_{f}$ and flexural strain ${\epsilon }_{f}$ for each specimen at all applied forces. The flexural elastic modulus ${E}_{f}$ is calculated following Eq. (1)

1 $${\sigma }_{f}=\frac{3FL}{2b{d}^{2}},{\epsilon }_{f}=\frac{6Dd}{{L}^{2}},{E}_{f}=\frac{{L}^{3}S}{4b{d}^{3}}$$

where *d*, *b*, and *L*, are the thickness, width, span length of the specimen, respectively. The load F at the fracture point and the deflection D at the center of the specimen is reported. Table 2 shows the specimen’s dimensions used for this test. The slope *S* is determined from the straight-line portion of the force–deflection curve. This test is done at the lab environmental condition of 22.6 °C and 36.7% relative humidity. The test method follows the ASTM D790-03^29^ standard flexure testing of plastics.

**Table 2:** The composite specimen’s dimensions used for the three-point bending moment test.

Sample number	Thickness *d* (mm)	Width *b* (mm)	Span *L* (mm)	Slop S
7	8.00	51.00	150.00	42.42
6	9.90	50.30	150.00	54.44

## Results and discussion

### Surface morphology characterization of the fibers and their composite

**Figure 9:**
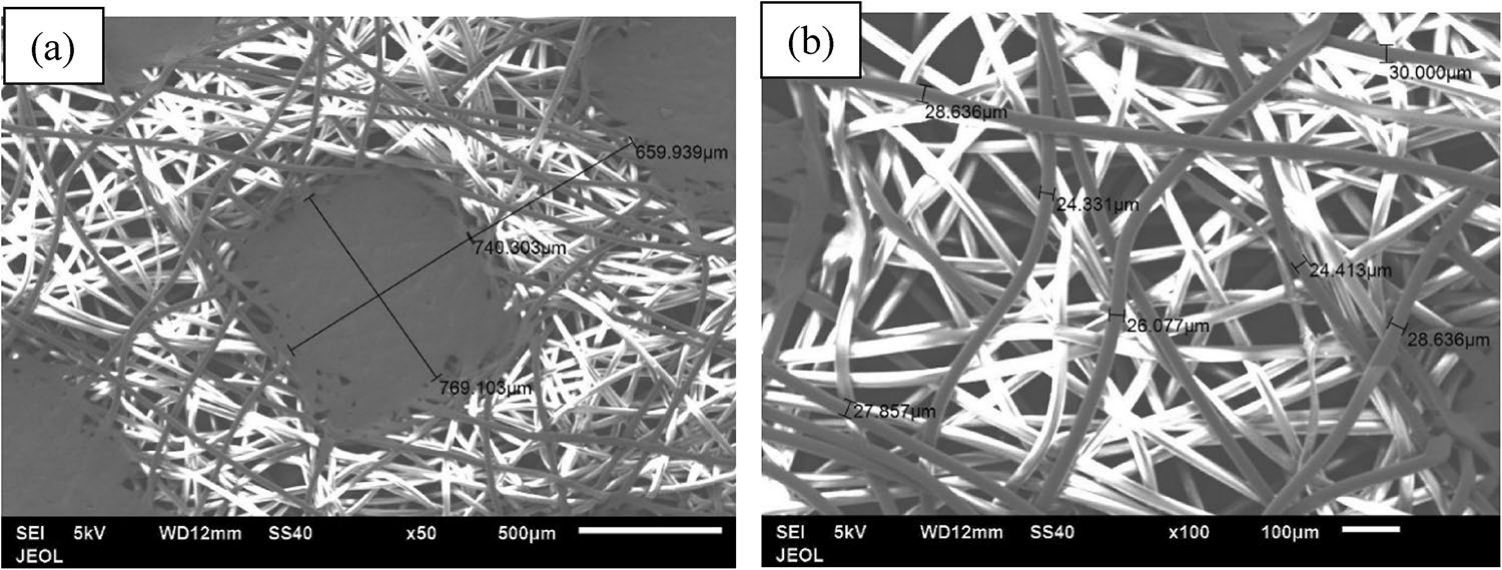
Fibers configuration of the first layer (blue) of the facemask; (**a**) showing the hollow square of the fibers and (**b**) the diameter of each fiber magnified 100 times.

**Figure 10:**
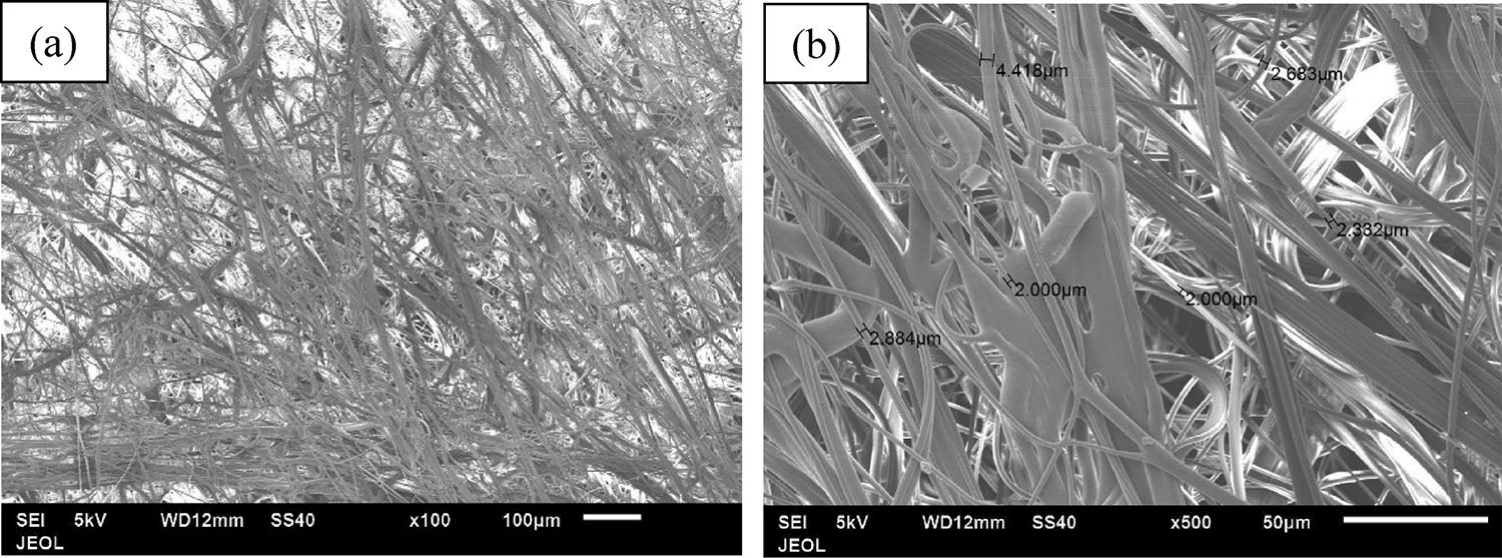
The middle layer fibers at two different magnifications; (**a**) at × 100 and (**b**) at × 500 showing the average size of the fibers.

**Figure 11:**
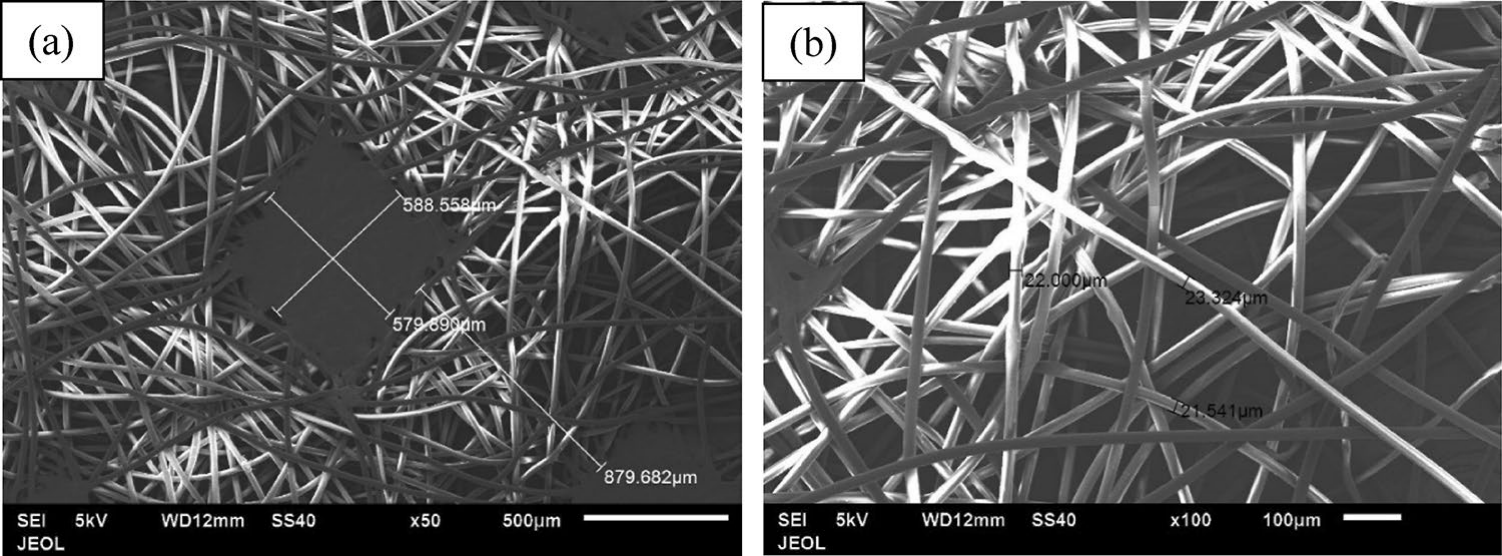
Fibers configuration of the third layer (white) of the facemask; (**a**) showing the hollow square of the fibers and (**b**) the diameter of each fiber magnified 100 times.

**Figure 12:**
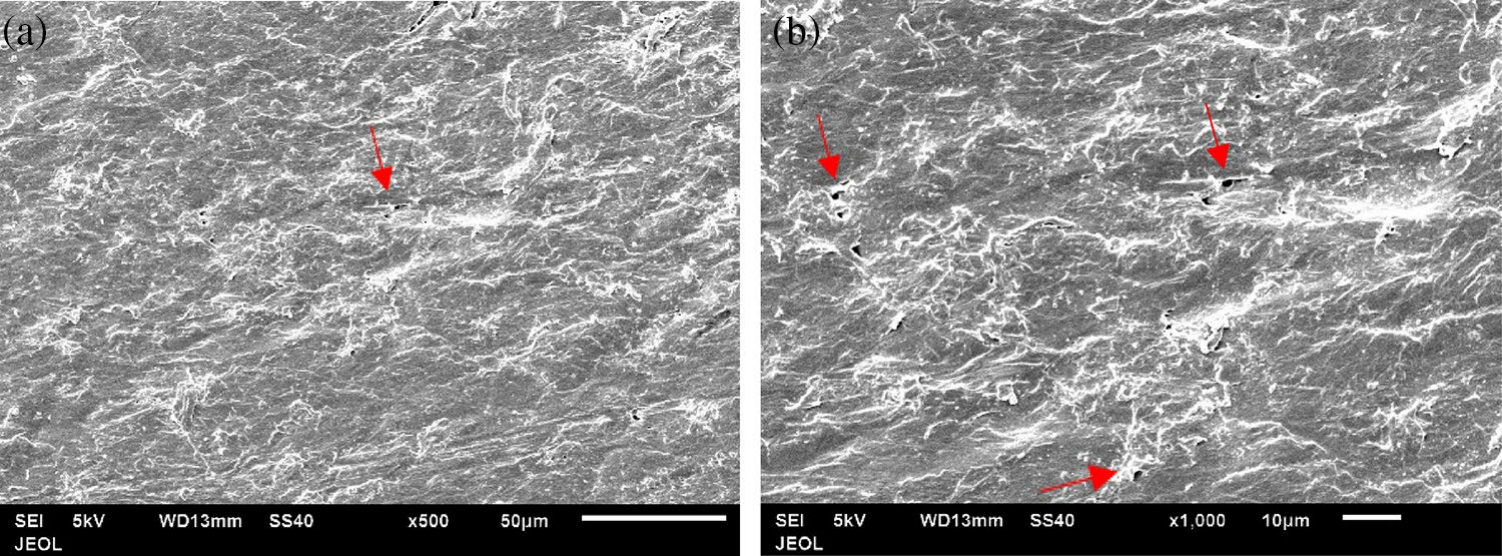
Magnified surface morphology of a composite (melted) of sample number 7 showing a very few pores marked by arrows, (**a**) at × 500 and (**b**) at × 1000.

Figure 9 shows the fibers of the first layer (blue layer), which is porous and consists of adjacent hollow squares configuration, formed by the fibers as seen in Fig. 9a. The average size of the hollow gap is about 769.103 × 740.303 μm and the width of the filled square side fibers is about 659.939 μm (Fig. 9a). The average diameter of the fibers, which make such squares, is 24.331–30.000 μm as seen in Fig. 9b. The middle layer fibers of the facemask are shown in Fig. 10 for two different magnifications of 100 and 500. The layer is porous also, but it does not have a specific configuration as the first layer. The average size of the fibers is 2.884–4.418 μm as seen in Fig. 10b. The third layer (white) has the same adjacent hollow square fibers similar to the first layer as shown in Fig. 11. However, the almost square hollow porous size of the layer is 579.89 × 588.56 μm and the width of the filled square side fibers is about 879.682 μm (Fig. 11a). The average size of each fiber is 21.54–23.32 μm as shown in Fig. 11b. Figure 12a, b shows the surface morphology of a melted piece of sample number 7 (without metal nose clips or elastic ear loops). It is noted that Fig. 12a, b has very few pores marked by arrows as dark black spots. This indicates that sample 7 and 6 are almost solid with a very few pores which make the thermal conductivity of such samples higher than the loose samples as will be seen in the thermal conductivity section.

### Thermal stability of the fibers and their composite

**Figure 13:**
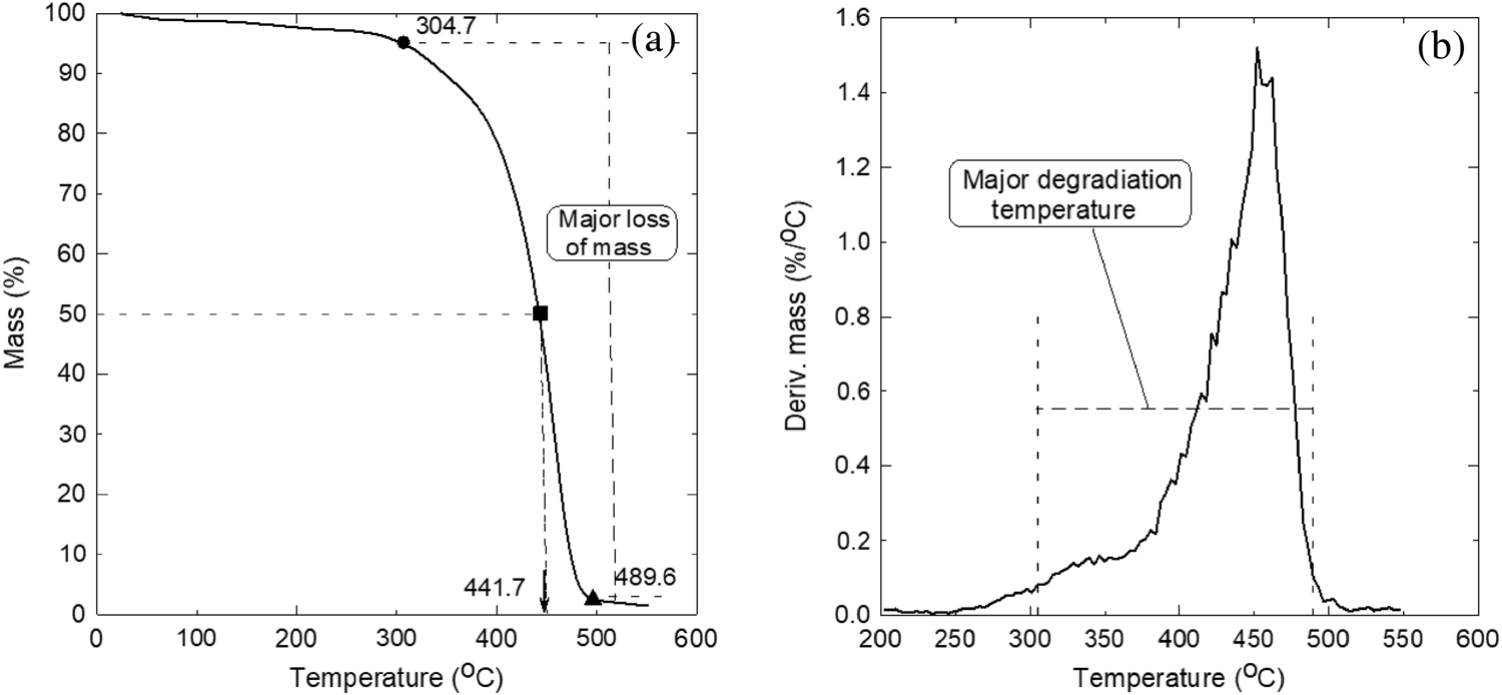
Thermal stability analysis of the elastic ear loops (sample number 5) showing the important points of degradation and decomposition, (**a**) thermogravimetric analysis (TGA) and (**b**) derivative thermogravimetric analysis (DTGA).

The thermal stability of the elastic ear loops as new thermal insulation materials is shown in Fig. 13a by using the TG analysis test. Only five percent of the mass is lost at the temperature range 25–304.7 °C, which could be due to the moisture content in the elastic ear loops. Therefore, the elastic ear loops are considered thermally stable up to 304.7 °C showing by ● symbol. Consequently, this point presents the start of degradation of the elastic ear loops. It should be noted that similar behavior was noticed by Asim et al.^30^; Marichelvam, Jawaid, and Asim^31^ and by Shahroze et al.^32^ for some agro wasted fiber materials. Symbol ■ presents another important point of decomposition, where the ear loops lose about 50% of their mass, which occurs at 441.7 °C. The third important decomposition point presented by ▲ symbol, where the material turned to a char since it lost about 97% of its mass at 489.6 °C. Figure 13b shows the major degradation temperature range of 304.7–489.6 °C, where the material lost almost all of its mass using the derivative thermogravimetric analysis (DTGA). This thermal analysis agrees very well with the finding obtained above when the oven was used to melt the three-layer facemasks at 230.9 °C with elastic ear loops, but the ear loops resist that as seen earlier in Fig. 4b. Figure 13a, b indeed ensures that elastic ear loops can stand a high degree of thermal stability up to 304.7 °C, which promote them to be used as thermal insulation materials developed from discarded waste materials.

**Figure 14:**
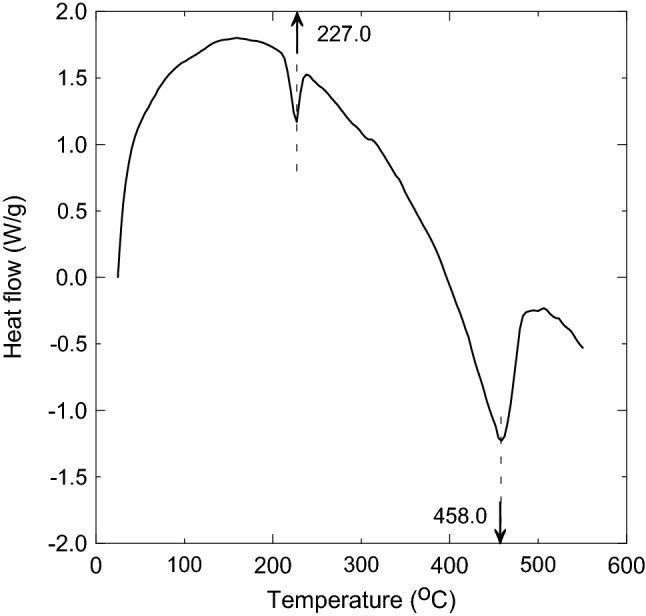
Elastic ear loops’differential scanning calorimetry (DSC) analysis showing the two transition peaks.

Figure 14 shows the differential scanning calorimetry (DSC) analysis up to 600 °C. This profile shows two broad endothermic transitions at 227 °C and 458 °C. In general, the transition may provide information about evaporation, melting, or decomposition of the fibers^33^. Therefore, the first transition (227 °C) may present the evaporation of moisture content in the elastic ear loops and the start of melting since at that temperature the loops are stable as shown in the TGA profile at Fig. 13a and lost only about 3% of their mass. The second transition (458 °C) presents a decomposition of the loops, where they lost about 71% of their mass.

**Figure 15:**
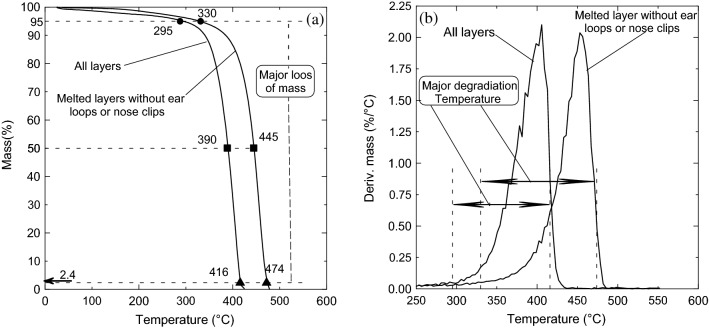
Thermal stability analysis of the three layer fibers and composite melted sample number 7 showing the important degradation and decomposition points, (**a**) thermogravimetric analysis (TGA) and (**b**) derivative thermogravimetric analysis (DTGA).

The thermal stability analysis is conducted for the mask’s three-layer fibers extracted from sample number 2 shown in Fig. 4b and for the composite melted sample number 7 shown in Fig. 5b. Figure 15a shows the thermogravimetric TGA profiles for both samples. Both samples are thermally stable up to 295 °C and 330 °C corresponding to the three-layer fibers and the composite, respectively. Both lose only 5% of their mass at these temperatures presented by ● symbol. The major loss of mass occurs between 295 and 416 °C for the three-layer and 330- 474 °C for the composite, where the degradation and decomposition occur. The critical point of decomposition, where the samples lost fifty percent of their mass at 390 °C and 445 °C for the three-layer and the composite, respectively. These points showing in the figure as ■. Both materials turned to a char (▲ symbol) at 416 °C and 474 °C for the three-layer and the composite, respectively since their remaining mass is about 2.4% of their original mass. The major degradation temperature for both samples is shown in Fig. 15b using the derivative thermogravimetric analysis (DTGA). This thermal analysis test shows that these materials can stand a high degree of thermal stability, which promotes them as new novel thermal insulation materials for buildings developed from discarded waste materials for possible replacement of synthetic or petrochemical insulation materials.

**Figure 16:**
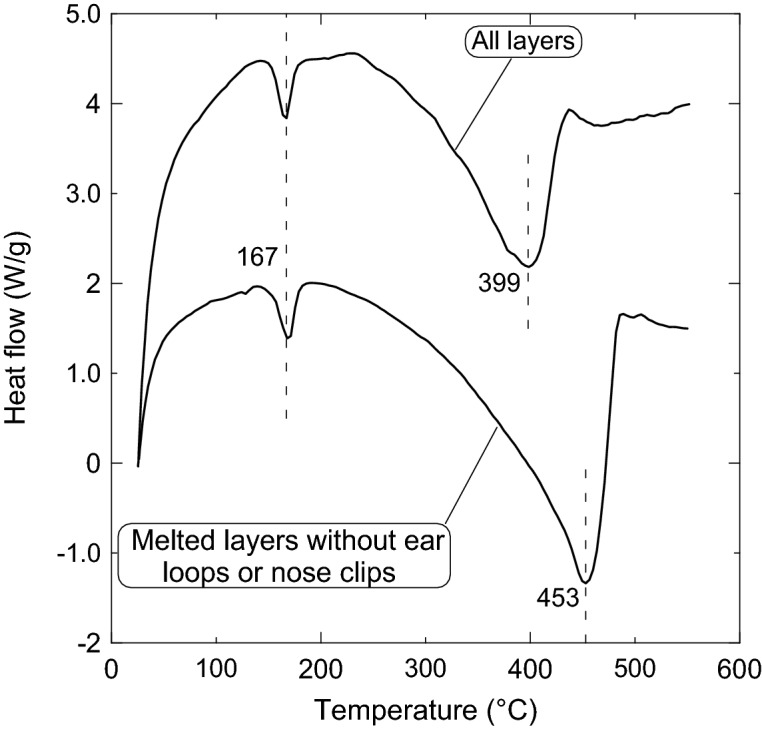
Differential scanning calorimetry (DSC) analysis showing the two transition peaks for both the three-layer and the composite samples.

Figure 16 shows the differential scanning calorimetry (DSC) analysis for both the three-layer and the composite samples. This figure shows that the first endothermic transition peak for both samples occurs at 167 °C, where the melting may start since both lost only about one or two percent of their original mass. This melted sample (# 7) has a comparable melting temperature with homo-polypropylene^34^, which has a melting temperature range of 160–166 °C.

However, the decomposition occurs at the second transition peak, where both samples lost about 65% of their mass. The temperatures of the second transition occur at 399 °C and 453 °C for the three-layer and for the composite sample, respectively. It should be noted that the DSC profile of both samples has a similar trend.

### Thermal conductivity measurements

**Figure 17:**
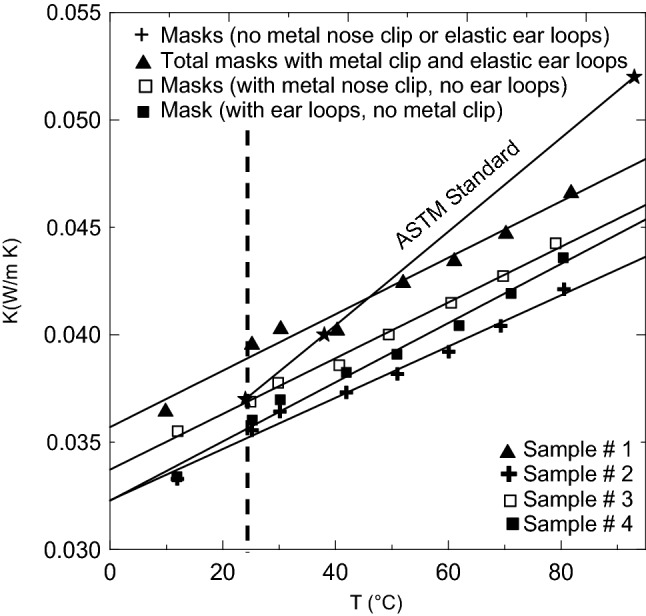
Thermal conductivity profiles at different temperatures for four various samples of loose facemasks (see Table 1 for more details).

Figure 17 shows the thermal conductivity profiles at different temperatures for a complete facemask with metal nose clip and elastic ear loops (▲symbols, sample # 1), facemasks with metal nose clip but without elastic ear loops (**□** symbols, sample # 3), facemasks with elastic ear loops but no metal nose clip (■ symbols, sample # 4), and facemasks without both metal nose clip and elastic ear loops (**+ **symbols, sample # 2). It is clear that removing both elastic ear loops and metal nose clip gives the best lower thermal conductivity at all temperatures. Consequently, since at room temperature of about 25 °C, presented by the dashed vertical line, the thermal conductivity of the four samples of used wasted facemasks is below 0.04 W/m K, which promotes them as a novel good cheap source of thermal insulation materials for buildings. The ASTM standard (* symbol) is also plotted for comparison with the current profiles to indeed indicate that using such wasted used facemasks is a promising new novel thermal insulation material. These new materials will be competitive to the synthetic and petrochemical insulation materials. It should be noted that the thermal conductivity coefficient increases as the density of the sample increase at fixed temperature and increases as the temperature increases for constant density. Solid lines present the linear best curve fit through the data for each sample on the form given by the following,

2 $$\left( {{\text{Thermal}}\,{\text{conductivity}}} \right) = {\text{A}} + {\text{B}}\left( {{\text{Temperature}}} \right)$$

where A and B are constants given in Table 3 with the coefficient of determination R^2^ for each data fit for each sample.

**Table 3:** Coefficients showing of Eq. (2) and the coefficient of determination for each sample.

	A	B	R^2^, %	Thermal conductivity coefficients at 25 °C
Sample 1, ▲	0.0357	0.000131	97.7	0.03963
Sample 2, +	0.0323	0.000119	98.7	0.03555
Sample 3, **□**	0.0337	0.00013	99.4	0.03688
Sample 4, ■	0.0323	0.000138	98.8	0.03602
Sample 5, ◇	0.0516	0.00022	97.6	0.05713
Sample 6, ●	0.0743	0.0006	98.5	0.08805
Sample 7, ○	0.0789	0.00032	99.3	0.08683

**Figure 18:**
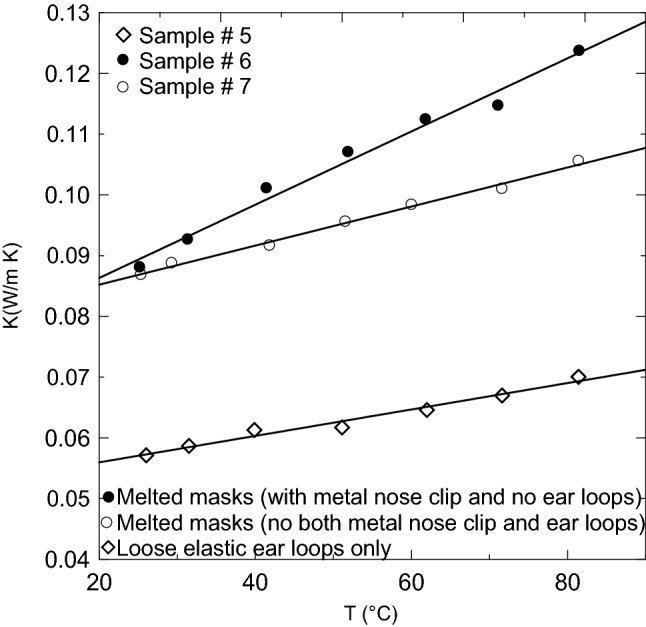
Thermal conductivity coefficient profiles at different temperatures for melted masks samples and for elastic ear loops.

Figure 18 shows the thermal conductivity coefficient profiles at different temperatures for the new novel composite (melted) masks’ materials and the loose elastic ear loops. It is (as expected) that the sample without a metal nose clip (○ sample # 7) gives a lower thermal conductivity than that with a metal nose clip (● sample # 6). Their thermal conductivity range at room temperature of 25 °C is 0.086–0.088 W/m K, respectively. This range is almost twice the values of loose masks shown in Fig. 17 since the melted ones lost most of the porous air channels inside the masks, which usually lower the thermal conductivity of the materials. The thermal conductivity profiles of loose elastic ear loops show a better lower thermal conductivity than the melted samples. Their thermal conductivity at room temperature is 0.057 W/m K. The solid lines shown in Fig. 18 present the linear best curve fit through the data following Eq. (2) and Table 3 gives the constants A, B, and the coefficient of determinations for samples 5, 6, and 7. Table 4 shows a comparison of the thermal conductivity coefficient of the current materials and some conventional and unconventional materials. All the current wasted facemask materials are better than polypropylene (PP)^34^ and polyethylene (PE)^34^. However, the unmelted samples have almost the same range of thermal conductivity coefficients as Rock wool^35^, Expanded Polystyrene^35^, phenol formaldehyde foam^36^, polyurethane foam^36^, and within the same range or better than the unconventional materials.

**Table 4:** Comparison between the thermal conductivity of the discarded facemask materials at different densities and other conventional and unconventional materials.

Materials	Thermal conductivity (W/mK)	References
Sample 1, ▲	0.03963	Current material
Sample 2, +	0.03555	Current material
Sample 3, **□**	0.03688	Current material
Sample 4, ■	0.03602	Current material
Sample 5, ◇	0.05713	Current material
Sample 6, ●	0.08805	Current material
Sample 7, ○	0.08683	Current material
Loose Eucalyptus Globulus leaves	0.0485–0.0560	^19^
Bound Eucalyptus Globulus leaves	0.0472–0.0599	^19^
Loose wheat straw	0.0432–0.0505	^19^
Bound wheat straw	0.0466–0.0569	^19^
Bagasse	0.0460–0.0550	^35^
Straw bale	0.0380–0.0670	^35^
Rice husk	0.0464–0.566	^35^
Corn cob	0.101	^35^
Date palm surface fibers	0.0475–0.0697	^20^
Hybrid (date palm tree surface fibers + Apple of Sodom fibers)	0.0423–0.0529	^17^
Rock wool	0.033–0.040	^35^
Expanded polystyrene	0.031–0.038	^35^
Polyurethane foam	0.025–0.035	^36^

### Sound absorption coefficient determination

**Figure 19:**
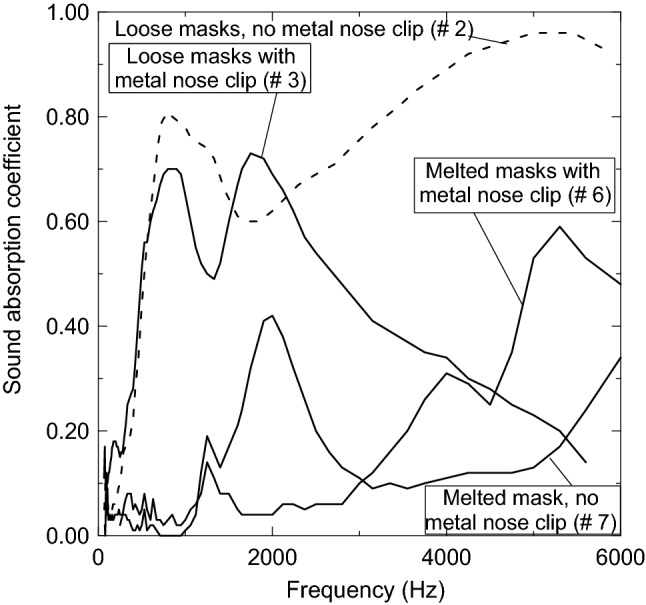
Sound-absorbing coefficients at wide range of frequency for loose and composite (melted) samples.

Figure 19 shows sound absorption coefficient profiles for loose facemasks with metal nose clip (sample # 3), loose facemasks without metal nose clip (sample # 2), melted facemasks with metal nose clip (sample # 6), and melted facemasks without metal nose clip (sample # 7). Sample number 2 without metal nose clip gives the best sound absorption coefficient since it is greater than 0.6 for a frequency greater than 580 Hz. Furthermore, it has a bell shape with a peak point of 0.81 at frequency 800 Hz, decreased up to 0.6 at frequency range 1700–1850 Hz, then increased to a maximum at frequency 5450 Hz. Loose sample number 2 with a metal nose clip has two bell shapes. The first bell shape reaches its peak of 0.7 at a frequency range of 800–900 Hz and the second peak of 0.73 is reached at 1750 Hz, and then decreases as the frequency increases. Therefore, sample number 2 is considered as the best sound-absorbing material for frequency greater than 580 Hz followed by sample number 3 for a frequency range 500–2650 Hz, where the sound-absorbing coefficient is greater than 0.51. These results for sound-absorbing coefficients for sample number 2 and 3 are comparable to that for ethylene–vinyl acetate and polypropylene mixture and with polystyrene and polypropylene mixture at different thicknesses with binders reported by Biskupicova et al.^37^. On the other hand, the melted facemasks do not give a promising sound-absorbing coefficient characteristic unless at a few high frequencies of 5000 Hz and 5300 Hz, where the coefficient is 0.53 and 0.59, respectively. These results are expected since the melted facemasks get rid of any porous channels, which lower the good characteristics of absorbing the sound. Sample number 2 and 3 with high sound-absorbing coefficient suggests using such waste materials, as a new source for sound-absorbing materials instead of synthetic and petrochemical acoustic materials.

### Mechanical properties of the composite samples

**Figure 20:**
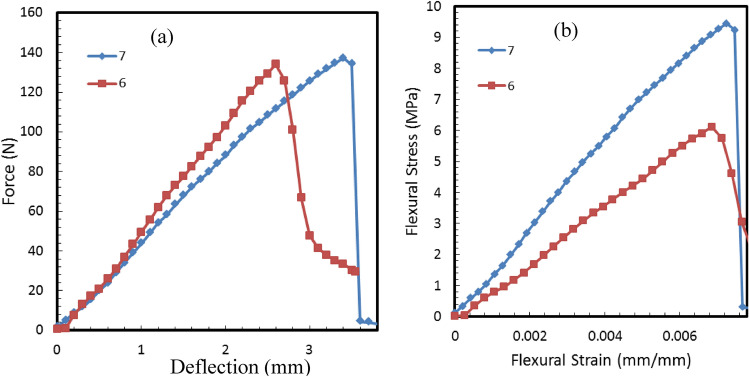
Bending tests for the composite (melted) samples 6 and 7, (**a**) load–deflection and (**b**) stress–strain profiles.

A three-point bending test is done for specimens extracted from the composite (melted) samples numbers 6 and 7 as specified in Table 2. The profiles of both load–deflection and the flexural stress–strain of these specimens are shown in Fig. 20a and b, respectively. The initial straight-line portion up to the elastic limit of the load–deflection curve (Fig. 20a) is used to calculate the slope *S *(*dF*/*dD*) for the two specimens. Table 5 shows the flexural modulus ${E}_{f}$, flexural Stress ${\sigma }_{f}$ and the flexural strain at flexural strength ${\epsilon }_{f}$, which is calculated at the end of the proportional limit of the elastic range on the load–deflection curve (Fig. 20a) when the curve starts to deviate from linearity^38^. It should be noted that the bending characteristics such as flexural modulus ${E}_{f}$, flexural Stress ${\sigma }_{f}$, and flexural strength ${\epsilon }_{f}$ have improved for higher density specimens, which agrees with the results obtained by Nguyen et al.^39^ and by Dukhan et al.^40^ using aluminum foam with polypropylene. It should be noted that flexural modulus ${E}_{f}$, shown in Table 5 is comparable to that of polypropylene^34^ (homopolymer and copolymer), which ranging between 1030 and 1310 MPa at different melting flow indexes.

**Table 5:** The flexural modulus ${E}_{f}$, flexural stress ${\sigma }_{f}$ and the flexural strain at flexural strength ${\epsilon }_{f}$ calculated at the end of the elastic limit of load- deflection curve for each specimen.

Specimen number	Slop S	Flexure Modulus (MPa), *E*_*f*_	Flexural Stress (MPa), *σ*_*f*_	Flexural strain at flexural strength *ϵ*_*f*_
7	42.42	1370.56	9.45	0.00725
6	54.44	941.12	6.12	0.00713

## Conclusion

Wasted loose facemasks can be used after heating at 120 °C for one hour as novel new thermal insulation for buildings. The thermal conductivity of the developed samples is 0.03963, 0.03688, 0.03602, 0.03555 W/m K at room environment temperature of about 25 °C using complete masks (sample # 1), without elastic ear loops (sample # 3), without metal nose clip (sample # 4), and without both of elastic ear loops and metal nose clip (sample # 2) respectively. The current studied materials have a better thermal conductivity coefficient than polypropylene (PP) and polyethylene (PE). The loose unmelted materials have comparable coefficients to Rock wool, Expanded Polystyrene, phenol formaldehyde foam, polyurethane foam and are within the same range or better than the unconventional materials. Acoustic characteristic showed that sample number 2 is the best among all samples since its sound-absorbing coefficient is above 0.6 for all frequency range and approaching 0.81 at 800 Hz and 0.96 at 5450 Hz. Followed by sample number 3, where the sound-absorbing coefficient is above 0.51 for the frequency range 500–2650 Hz. The loose facemasks, elastic ear loops, and composite (melted) facemasks are found to be thermally stable up to 295 °C, 304.7 °C, and 330.0 °C, respectively. The composite specimens can stand high values of flexural stress, flexural strain, and flexural elastic modulus. Using these wasted discarded materials for both thermal insulation and sound-absorbing in buildings will lower the environmental impact and solve the world great problem of getting rid of such wasted materials.
